# Characterization of the Vaginal Microbiota of Ewes and Cows Reveals a Unique Microbiota with Low Levels of Lactobacilli and Near-Neutral pH

**DOI:** 10.3389/fvets.2014.00019

**Published:** 2014-10-15

**Authors:** Jeffrey D. Swartz, Medora Lachman, Kelsey Westveer, Thomas O’Neill, Thomas Geary, Rodney W. Kott, James G. Berardinelli, Patrick G. Hatfield, Jennifer M. Thomson, Andy Roberts, Carl J. Yeoman

**Affiliations:** ^1^Department of Animal and Range Sciences, Montana State University, Bozeman, MT, USA; ^2^United States Department of Agriculture-Agricultural Research Service, Miles City, MT, USA

**Keywords:** vaginal microbiota, vaginal pH, *Lactobacillus*, *Aggregatibacter*, *Streptobacillus*

## Abstract

Although a number of common reproductive disorders in livestock involve bacterial infection, very little is known about their normal vaginal microbiota. Therefore, we sought to determine the species composition of sheep and cattle vaginal microbiota. Twenty Rambouillet ewes and twenty crossbred cows varying in age and reproductive status were sampled by ectocervicovaginal lavage. We amplified and sequenced the V3–V4 region of the 16S ribosomal RNA (rRNA) contents yielding a total of 907,667 high-quality reads. Good’s Coverage estimates indicated that we obtained data on 98 ± 0.01% of the total microbial genera present in each sample. Cow and ewe vaginal microbiota displayed few differences. Cow microbiota exhibited greater (*P* ≤ 0.05) α-diversity compared to the ewe microbiota. Both livestock species differed (*P* ≤ 0.05) from all previously reported vaginal communities. While bacteria were numerically dominant, Archaea were detected in 95% of cow and ewe samples, mainly of the order Desulfurococcales. Both ewes and cows were predominately colonized by the bacterial phyla Bacteroidetes, Fusobacteria, and Proteobacteria. The most abundant genera were *Aggregatibacter* spp., and *Streptobacillus* spp. *Lactobacillus* spp. were detected in 80% of ewe and 90% of cow samples, but only at very low abundances. Bacteria previously described from culture-based studies as common to the cow and ewe vaginal tract, except for *Escherichia*, were variably present, and only in low abundance. Ewe and cow pH differed (*P* ≤ 0.05), with means (±SD) of 6.7 ± 0.38 and 7.3 ± 0.63, respectively. In conclusion, 16S rRNA sequencing of cow and ewe vaginal ectocervicovaginal lavages showed that cow and ewe vaginal microbiota differ from culture-led results, revealing a microbiota distinct from previously described vaginal ecosystems.

## Introduction

The human vaginal microbiota is most often dominated by lactobacilli (1). In this system, the lactobacilli are considered important to vaginal homeostasis through their production of lactate, which maintains a low vaginal pH (pH < 4.5) that is inhibitory to many vaginal pathogens (2). A reduction in vaginal lactobacilli, which is typically accompanied by an elevated vaginal pH, is a common feature of bacterial vaginosis (BV), the most common disorder among reproductive-aged women (3). BV is of significant concern, particularly due to its exacerbation of the risks of pre-term birth and spontaneous abortion (4). Given the potential importance of vaginal lactobacilli in diminishing the risks of pregnancy-related complications, it is interesting to note that the vaginal microbiota of some humans (1) and all primates (5) do not display the same *Lactobacillus*-dominated vaginal ecosystems. Similarly, the few culture-based studies that have been conducted on livestock have reported *Lactobacillus* spp. at lower abundances than other microbial genera in both the cow and ewe vagina (6–8). *Enterococcus* spp., *Staphylococcus* spp., and *Streptococcus* spp. are more commonly isolated from the cow vagina (6, 7, 9, 10), while *Bacillus* spp., *Corynebacterium* spp., *Escherichia* spp., *Staphylococcus* spp., and *Streptococcus* spp. are commonly isolated from the ewe vagina (11–14). To date, no studies have been reported that have utilized culture-independent 16S ribosomal RNA (rRNA) sequencing of the cow or ewe vaginal microbiota. Previous applications of 16S rRNA sequencing techniques have revealed a much greater and historically unrealized diversity of microbiota in various ecosystems (15–17). Other studies have indicated that culture-based approaches may emphasize the rarer members of communities and often miss those microbes that are more abundant (16). The complete reliance on culture has thereby left the true microbial diversity of the cow and ewe vagina hereinto undetermined and the scarcity of lactobacilli uncertain. Therefore, the objective of this study was to elucidate the microbiota present in cow and ewe vaginas using culture-independent 16S rRNA sequencing technology and determine how the composition of the livestock vaginal microbiota compares to other well-described vaginal microbial communities that have been similarly defined in humans and non-human primates (5). This information will provide a basis to evaluate the livestock vaginal microbiota’s potential roles in affecting vaginal health, reproductive outcomes, and perinatal morbidities. Specifically, we seek to develop the necessary background information required to examine the hypotheses that: (i) specific livestock vaginal communities increase or decrease the risks of acquisition of livestock venereal pathogens alike *Brucella ovis*, *Campylobacter fetus*, *Helicobacter trogontum*, or *Arcobacter cryaerophilus*; (ii) that the vaginal microbiota are the first colonizers of neonatal animals, as has been shown for conventionally born human neonates (18), and, due to the relative importance of this early microbiota (19), directly influence perinatal health and performance; and (iii) that specific vaginal microbial communities of cattle and sheep impact reproductive outcomes, including spontaneous abortion. Well-described infectious agents of the livestock vagina have already been associated with placentitis, abortion, infertility, and the birth of debilitated offspring (20–23). Evidence from human studies indicates that vaginal dysbiosis increases the risks of acquisition and shedding of sexually transmitted infections (24–27), while, a healthy vaginal microbiota is more resistant to venereal infection. Additionally, given the importance of pH to human vaginal health, livestock vaginal pH was also investigated.

## Materials and Methods

### Ethics statement

Animal care and use protocols were approved by the Montana State University Agricultural Animal Care and Use (AACUC) committee under protocol number 2012-AA07 dated 09/20/2012.

### Sampling

Vaginal lavages were collected from 20 Rambouillet ewes and 20 crossbred beef cows of varied breeding method and pregnancy status (Table 1). Sampled ewes were being maintained at the Bozeman Agricultural Research and Teaching farm, Bozeman, MT, USA, while cows were sampled 286 miles away at Fort Keogh, Miles City, MT, USA. The cows and ewes had never been cohabitated. Samples were collected by injecting 25 ml (ewes) or 50 ml (cows) of sterile saline into the vaginal tract via sterile catheter tubes attached to luer lock 60 ml syringes. Saline was injected in a continuous stream toward the cervix, aspirated 3–5 times, transferred to a sterile 15 ml falcon tube, and stored at −20°C. One milliliter of sample was separated for pH determination. For DNA extraction, up to 4.5 ml of sample was centrifuged for 5 min at 20,000 × *g* and 4°C.

**Table 1:** **Pregnancy status and age of animals used in this trial**.

Species	Animal ID	Pregnancy status^a^	Age
Ewe	J0441	Not mated^b^	2
	J1013	Not mated^b^	1
	J0459	Not mated^b^	2
	J9013	Not mated^b^	3
	J8002	Not mated^b^	4
	J1445	<48 h Since first mounting	1
	J8030	<48 h Since first mounting	4
	J8436	<48 h Since first mounting	4
	J8456	<48 h Since first mounting	4
	J8487	<48 h Since first mounting	4
	J9444	Open^a^	3
	J9017	Open^a^	3
	J0025	Open^a^	2
	J0037	Open^a^	2
	J0447	Pregnant^a^	2
	J9005	Pregnant^a^	3
	J9014	Pregnant^a^	3
	J9404	Pregnant^a^	3
	J9445	Pregnant	3
	J8029	Pregnant^a^	4
Cow	C09925	Not mated^b^	2
	C10742	Not mated^b^	1
	C09836	Not mated^b^	2
	C10896	Artificially inseminated	1
	C06901	Artificially inseminated	2
	C09808	Artificially inseminated	2
	C08853	Embryo transfer recipient	2
	C09891	Embryo transfer recipient	2
	C10840	Embryo transfer recipient	1
	C99842	Open^a^	3
	C09727	Open^a^	2
	C10687	Open^a^	1
	C05E16	Open^a^	2
	C09703	Open^a^	2
	C99791	Pregnant^a^	3
	C10E16	Pregnant^a^	1
	C05X77	Pregnant^a^	2
	C06981	Pregnant^a^	2
	C06988	Pregnant^a^	2
	C02851	Pregnant^a^	2

*^a^Pregnancy status at time of sample acquisition*.

*^b^Not mated in the current season*.

### pH analyses

A 1 ml subsample from each lavage was assessed for pH using a Ryan 520A pH meter fitted with a ROSS Ultra Electrode (Thermo Scientific, Waltham, MA, USA). All animals were used for analysis of pH except ewes sampled before breeding (Table 1), due to the lavage potentially containing phosphate buffer (Table 1). Mean, SD, boxplots, Shapiro–Wilk test of normality, and two-sided Wilcoxon rank sum test were calculated using R (28).

### Sequencing microbiota

Pellets obtained from up to 4.5 ml of lavage were extracted using MoBio PowerSoil DNA Isolation kits following manufacturer instructions, except a 2 min bead-beating step was used instead of a 10 min vortex. Variable regions three and four of the 16S rRNA genes were amplified using custom primers that included indexes to identify samples after sequencing. The PCR reaction ran for 30 cycles at 94°C for 20 s, 52°C for 30 s, and 72°C for 45 s, using barcoded primers [SeqF(1–8) 5′-AATGATACGGCGACCACCGAGATCTACAC(adaptor)-Index2(1 of 8 different 8 nt codes used to distinguish among samples when pooled for sequencing)-TATGGTAATT(sequencing primer pad)-AT(linker)-CCTACGGGAGGCAGCAG(341f primer)-3′ and SeqR (1-12) 5′-CAAGCAGAAGACGGCATACGAGAT(adaptor)-Index1 (1 of 12 different 8 nt codes used to distinguish among samples when pooled for sequencing)-AGTCAGTCAG(sequencing primer pad)-CC(linker)-GGACTACHVGGGTWTCTAAT(806r primer-3′]. Amplicons were quantified using an Agilent 2200 tape station (Agilent, Santa Clara, CA, USA) and pooled at an equimolar concentration. Pooled amplicons were purified from residual PCR reagents and non-specific amplification products in an agarose gel using a QIAquick gel extraction kit (Qiagen, Valencia, CA, USA) following manufacturers instructions. Purified and pooled amplicons were subsequently quantified using a KAPA Syber quantification kit (KAPABiosystems, Wilmington, MA, USA) as per manufacturer instructions. Purified, quantified, and pooled amplicons were mixed with 5–10% of an equimolar concentration of PhiX and sequenced at 12.5 pM. Sequencing was performed with an Illumina MiSeq using paired-end 2 × 250 nucleotide (nt) dual-index sequencing. Custom primers (R1 5′-CCTACGGGAGGCAGCAG-3′, R2 5′-AGTCAGTCAGCCGGACTAC-3′, and Index 5′-GTAGTCCGGCTGACTGACT-3′) were used for sequencing and indexing. Raw sequence data were deposited within the short read archive under experiment accession number SRX708102, and processed data were available from the researchers upon request.

### Data analyses

The resulting paired-end 16S rRNA gene reads were assembled using the “make.contigs” command in mothur^1^ (29). Any base call disagreement in the overlapping portions of the paired reads was ascribed to the base with a higher quality score (“deltaq = 1”) or called “N” if both nucleotides were below Q20. Assembled sequences were then pre-processed to remove low quality and chimeric sequence data. Sequences were removed if they were shorter than 400 nt, had a homopolymeric sequence greater than 10 base calls, had more than two ambiguous base calls (1 per 200 nt), or were found to be chimeric sequences by UCHIME (30). Each of the resulting sequence data sets were subsampled to 5,000 reads to enable direct comparison, as per Schloss et al. (31). Reads were preclustered as per Huse et al. (32) to reduce the influence of sequence error. The resulting data were separately clustered to form operational taxonomic units (OTUs) using mothur’s farthest neighbor approach at 95% sequence identity, or taxonomically assessed using mothur’s implementation of the naïve Bayesian classifier, RDP Classifier (33). OTUs representing < 0.1% of the total 16S rRNA reads in a given sample were eliminated as noise, as this has previously been shown to be the approximate level of noise from Illumina sequencing of complex communities (34). Taxonomic assignments were considered supported if bootstrapping values were greater than 70%. Microbial 16S rRNA gene composition and diversity were compared among samples, as well as to sequences from human and primate vaginal systems, using multivariate statistical approaches provided by mothur for measures of γ-, and α-diversity, and using the vegan package of R (35) for β-diversity. Richness measures included genera observed and Chao1 estimates (36) of total genera richness. These measures were derived from genus-level taxonomic classifications due to the low bootstrapping support offered to the majority of sub-genus-level classifications. Diversity measures were determined using Shannon’s diversity index. Normality of data was tested using the Shapiro–Wilk test (37), and significance was determined using a two-sample *t*-test or Wilcoxon Mann–Whitney test for normally and non-normally distributed data, respectively. Heatmaps were constructed using gplots^2^ in R with taxonomic data. Within and across microbial communities, inter-individual, and inter-species similarities were determined by pairwise measurements of Bray–Curtis dissimilarity, with significance determined by analysis of similarities (ANOSIM).

## Results

### Sequencing overview

Twenty Rambouillet ewes and twenty crossbred cows were sampled to investigate the composition of livestock vaginal microbiota. Samples were used to generate deep V3-V4 16S rRNA gene profiles. A total of 907,667 high-quality reads were obtained following processing, and samples were randomly subsampled to 5,000 reads for direct comparison. Good’s Coverage estimates (38) were not different among cattle and sheep, indicating that this approach obtained data on 98 ± 0.01% of the total microbial genera present in each sample. Consistently, rarefaction curves appear to be trending toward an asymptote (Figure S1 in Supplementary Material).

### α-Diversity

Unless otherwise stated, analyses were carried out at genus-level resolution due to low bootstrapping support for species-level taxonomic designations. A greater number of genera (comparative *t*-test; *P* ≤ 0.05) were detected in cow vaginal samples compared to ewe samples, with 302 ± 83 and 220 ± 102 genera, respectively. A few outliers existed for each host species as seen in the rarefaction curves (Figure S1 in Supplementary Material), however these were not significantly associated with mating, fertilization method, or reproductive status (all comparative *t*-tests *P* > 0.05). Chao1 predicted that there may have been more (comparative *t*-test; *P* ≤ 0.05) genera present in cow than ewe vaginal samples, with 394 ± 77 and 310 ± 103 total genera predicted, respectively. However, most genera were observed to be present at very low relative abundances with just 14 ± 4 and 11 ± 4 genera representing greater than 1% of the reads, and 90 ± 41 and 57 ± 45 representing >0.1% of the reads, respectively. Measures of diversity (richness and evenness) were normally distributed (Shapiro–Wilk *W* = 0.92; *P* > 0.05) for ewes but not cows (*W* = 0.9; *P* ≤ 0.05).

**Figure 1:**
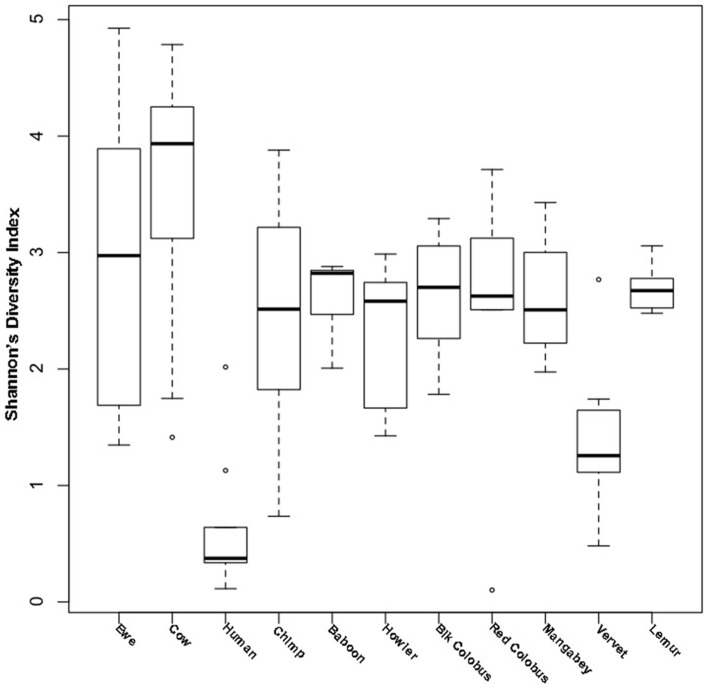
**Shannon’s diversity of livestock vaginal microbiota as compared to humans and non-human primates**. Boxplots showing the median, quartiles, and extremities of Shannon’s diversity index values calculated for individual ewes, cows, humans, and non-human primates compared in this study.

Diversity was measured with Shannon’s diversity index, which indicated low to moderate diversity communities for both ewes and cows at 2.87 ± 1.16 and 3.64 ± 0.96, respectively. Cow vaginal microbiota exhibited greater diversity as measured with Shannon’s diversity index than ewes (Wilcoxon; *P* ≤ 0.05), humans (Wilcoxon; *P* ≤ 0.05), and all non-human primates (Wilcoxon; *P* ≤ 0.05). However, the diversity of ewe vaginal microbiota was not significantly different (*P* > 0.05) from most non-human primates, although was still more diverse (Wilcoxon; *P* ≤ 0.05) than the human vagina (Table S1 in Supplementary Material and Figure 1).

### β-Diversity

Due to low bootstrapping support for species-level taxonomic designations, we employed an OTU approach (described in Section “Materials and Methods”) to compare the compositions of cow and ewe vaginal microbiota. A small but significant difference was determined between the OTU compositions of cow and ewe vaginal microbiota (ANOSIM *R* = 0.11, *P* ≤ 0.05). However, no significant difference was seen between the cow and ewe vaginal microbiota when comparing genus-level taxonomic data (ANOSIM R = 0.07, *P* > 0.05) indicating the small differences observed resulted from sub-genus-level distinctions. No difference was seen in the overall composition with age, even when stratified by host species (ANOSIM R < 0, *P* > 0.05). Method of fertilization (AI vs. conventional; ANOSIM *R* = 0.02, *P* > 0.05) or embryo transplantation in cows (ANOSIM *R* = 0.04, *P* > 0.05) did not lead to significant overall differences in vaginal microbiota. At the time of sampling, unmated ewes and cows did not differ from mated (ANOSIM *R* < 0.2, *P* > 0.05) or from pregnant animals (ANOSIM *R* < 0.07, *P* > 0.05). Unmated ewes were not significantly different from recently (<48 h) mated animals (ANOSIM *R* = 0.2, *P* = 0.09). Conventionally bred cows and ewes (both ANOSIM *R* < 0, *P* > 0.05) also did not differ among pregnant and mated but open individuals.

**Figure 2:**
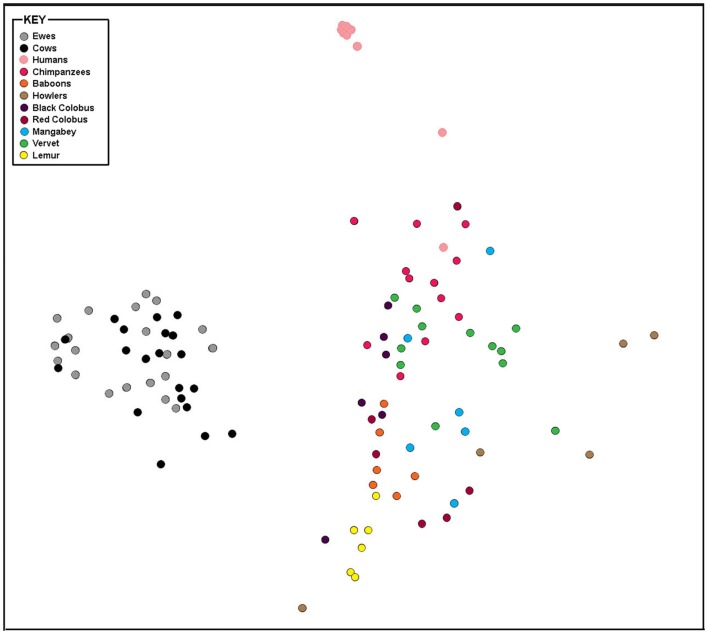
**Bray–Curtis relationship among vaginal microbiota of livestock, humans, and non-human primates**. Non-metric multi-dimensional scaling plot of Bray–Curtis dissimilarity measures of vaginal microbiota determined from individual ewes, cows, humans, and non-human primates compared in this study.

The taxonomic composition inferred from 16S rRNA gene sequence data was compared using genus-level resolution to human and non-human primate vaginal microbiota determined by Yildirim et al. (5). Continued use of genus-level resolution was further necessitated by the limited overlap in the regions on the 16S rRNA gene molecule sequenced in this study and by Yildirim et al. (5). RDP Classifier genus-level taxonomy was ordinated by non-metric multi-dimensional scaling (NMDS) using Bray–Curtis dissimilarities (Figure 2). At this level of taxonomic resolution, the host species-specificity of vaginal microbiota among many of the non-human primate species, as previously reported by Yildirim et al. (5), had eroded. However, alike human vaginal microbiota, ewe and cow vaginal microbiota were distinct. Both differed significantly from humans (*R* = 1, *P* ≤ 0.05) and non-human primate host species (*R* > 0.7, *P* ≤ 0.05).

### General taxonomic compositional traits

**Figure 3:**
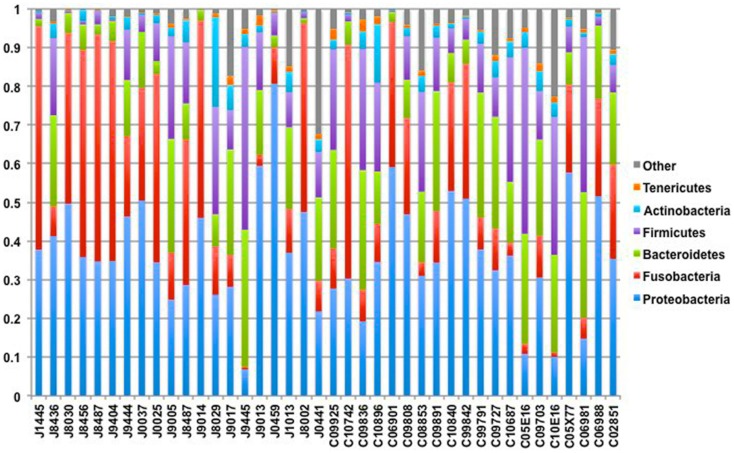
**Phylum-level composition among livestock vaginal microbiota**. Bar chart showing the proportional distribution of the six most abundant phyla.

**Figure 4:**
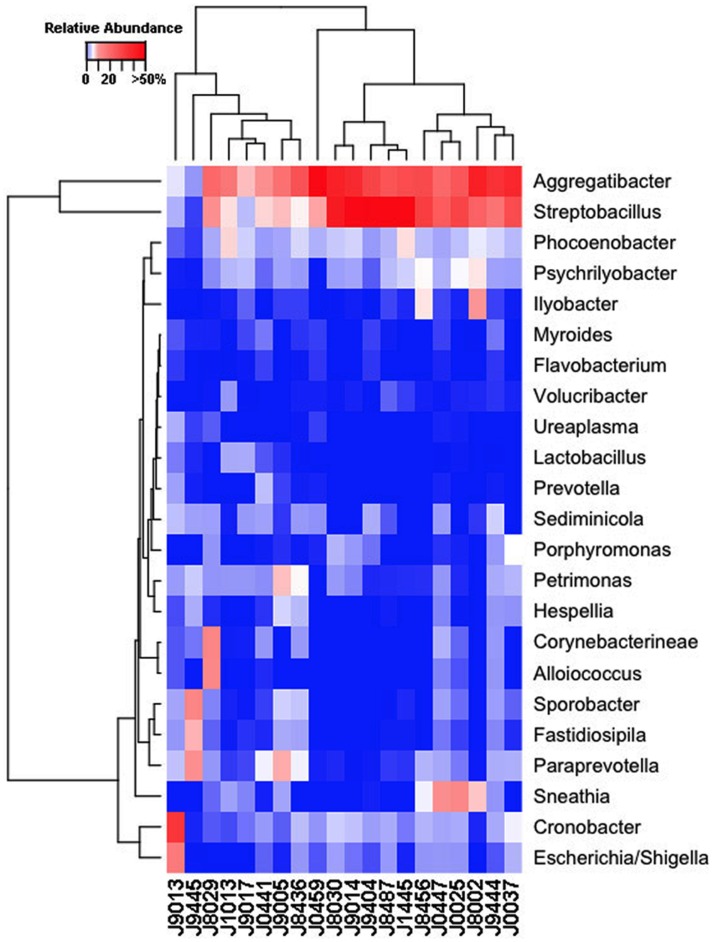
**Genus-level composition among ewe vaginal microbiota**. Heat map showing the relative abundances of the most abundant genera identified in individual ewe vaginal microbiota. Color breaks in heatmap are adjusted to show genera seen at <1% (blue shades), 1–10% (white shades), and >10% (red shades) relative 16S rRNA gene abundance.

**Figure 5:**
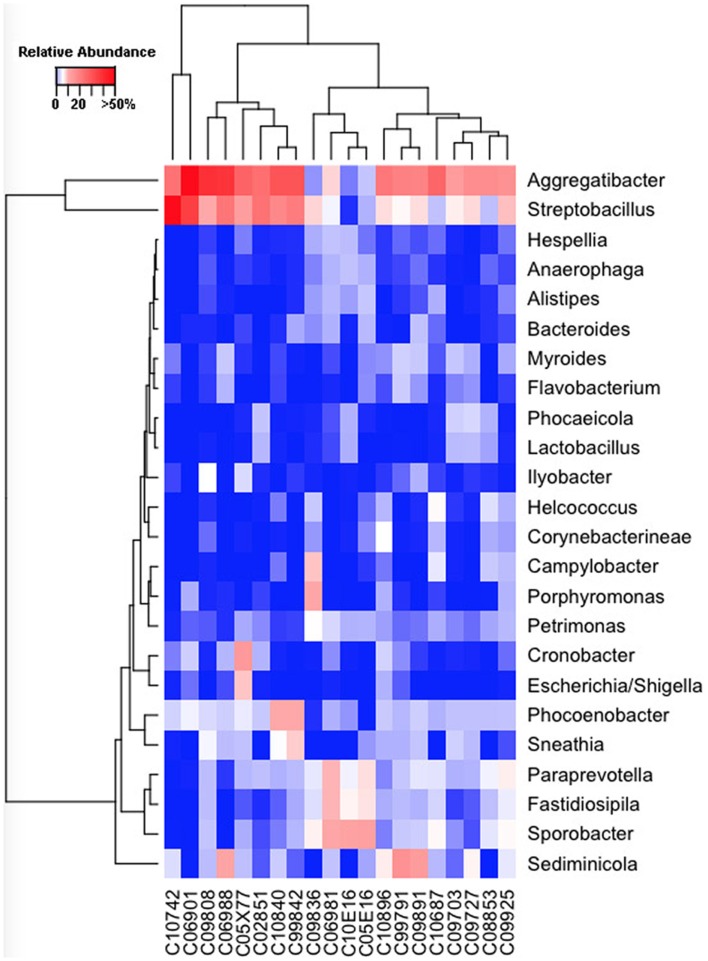
**Genus-level composition among cow vaginal microbiota**. Heat map showing the relative abundances of the most abundant genera identified in individual cow vaginal microbiota. Color breaks in heatmap are adjusted to show genera seen at <1% (blue shades), 1–10% (white shades), and >10% (red shades) relative 16S rRNA gene abundance.

Our sequencing approach enabled the detection of both bacterial and archaeal microbiota. Bacteria were numerically dominant, representing 98.7 ± 0.02% of 16S rRNA reads in all samples. However, Archaea were detected in 95% (19/20) of vaginal samples collected from both cows and ewes. Archaeal reads were largely assigned to members of the order Desulfurococcales, occurring in 95% of cow and 85% of ewe samples. The bacterial community was most commonly dominated by members of the Proteobacteria (almost exclusively γ-proteobacteria), Fusobacteria, and Bacteroidetes phyla in both ewes and cows (Figure 3). *Aggregatibacter* spp. and *Streptobacillus* spp. were typically the most abundant genera in both ewes and cows, while various other genera were observed (Figures 4 and 5). Lactobacilli were common, being detected in 80% (*n* = 16/20) of ewe, and 90% (*n* = 18/20) of cow vaginal samples. However, lactobacilli were always found at a low relative abundance (0.36 ± 0.66 and 0.53 ± 0.65%) of the total 16S rRNA gene population determined from both cattle and ewe vaginal samples, respectively. Assignable *Lactobacillus* species varied among individual animals and were often heterogeneous within samples (Table 2). Species often described in human vaginal microbiota, particularly those defined by Ravel et al. (1) as community state type (CST) IV, and often associated with BV, were observed in ewes and cows. These include *Sneathia* spp. that were observed in 90% of samples from both ewes and cows at 2.4 ± 4.0 and 1.9 ± 2.3% of the total microbiota, respectively, and *Prevotella* spp. that were observed in 65% of ewes at 0.5 ± 0.9% of the total microbiota. *Paraprevotella* spp., a distinct but closely related genera to *Prevotella* were also observed among both ewe and cow vaginal microbiota.

**Table 2:** **Lactobacilli identified in cow and ewe ectocervicovaginal lavages**.

Lactobacilli	Number of ewes	Number of cows
*Lactobacillus acetotolerans*	1	0
*Lactobacillus acidophilus*	1	0
*Lactobacillus amylolyticus*	0	1
*Lactobacillus animalis*	1	0
*Lactobacillus aviarius*	1	1
*Lactobacillus capillatus*	0	2
*Lactobacillus delbrueckii*	1	1
*Lactobacillus equi*	4	4
*Lactobacillus equigenerosi*	3	1
*Lactobacillus hayakitensis*	6	1
*Lactobacillus helveticus*	0	1
*Lactobacillus intestinalis*	1	0
*Lactobacillus kalixensis*	0	1
*Lactobacillus kunkeei*	1	0
*Lactobacillus mali*	1	0
*Lactobacillus oligofermentans*	1	0
*Lactobacillus* spp.^a^	12	17

*^a^Not unambiguously assignable to any single *L*. spp*.

### Livestock vagina maintains a near-neutral pH

Consistent with low *Lactobacillus* spp. abundance in vaginal microbiota, vaginal pH was near-neutral in both cows and ewes. Cow C99842 was noted to have a yellow sample, possibly containing urine. Significance of results was not affected by inclusion or exclusion of this value, except that Shapiro–Wilk test reported that cow pH was not normal (*P* ≤ 0.05) with C99842 and was normal (*P* > 0.05) following its removal. Ewe and cow pH differed (*P* ≤ 0.05) as determined by Welches and Wilcoxon two-sample tests before and after removal of C99842. The pH means (±SD) were 6.7 ± 0.38 and 7.3 ± 0.63, and ewes ranged from 5.6 to 7.1, and cows from 6.5 to 8.7.

## Discussion

Despite well-described roles in human reproduction and perinatal health, the vaginal microbiota of very few non-human hosts has been described to date. Thereby their relationships with reproductive outcomes and perinatal morbidity remain to be explored. The human vagina is most commonly dominated by *Lactobacillus* spp. during their reproductive years (1), however, a recent study of non-human primates (5) has revealed this to be unique among the primate order. Our findings herein indicate the uniqueness of the human vaginal microbiome extends further into Mammalia. *Aggregatibacter* spp., *Streptobacillus* spp., *Cronobacter* spp., *Phocoenobacter* spp., and *Psychrilyobacter* spp. were found to be the predominant bacterial genera of the ewe vaginal tract. While, *Aggregatibacter* spp., *Streptobacillus* spp., *Phocoenobacter* spp., *Sediminicola* spp., and *Sporobacter* spp. were the major genera in cow samples. Our results contrast with preceding culture-based studies that have more commonly isolated genera such as *Bacillus* spp., *Staphylococcus* spp., and *Streptococcus* spp., from both cow and ewe vaginas as well as *Corynebacterium* spp., and *Escherichia* spp., from the ewe vagina and *Enterococcus* spp. from the cow vagina (6, 7, 9–13, 39). These bacterial genera described in culture-based studies were often detected in our samples but typically with low relative abundances. *Escherichia* spp. were detected in all but one ewe (19/20; Figure 4), while the rest of the bacteria suggested to be prevalent by culture-based studies were seen at very low abundances in 3–11 of the ewes examined. Similarly, those bacteria commonly cultured from the cow vagina were detected at very low abundance in 0–11 of the 20 cows sampled in this study. A recent 16S rRNA sequencing study of Holstein cow uteri reported the presence of the same bacterial phyla as we observed in the cow and ewe vagina, specifically, Actinobacteria, Bacteroidetes, Firmicutes, Fusobacteria, Proteobacteria, and Tenericutes (40). The same study also reported many genera that we detected as prevalent in the ewe and cow vaginal samples, including *Escherichia* spp., *Lactobacillus* spp., *Porphyromonas* spp., *Prevotella* spp., *Sneathia* spp., *Streptobacillus* spp., and *Ureaplasma* spp. and other genera that were observed specifically in the cow vaginal samples, including *Alistipes* spp., *Bacteroides* spp., *Campylobacter* spp., and *Helcococcus* spp. (40).

Studies exploring microbial communities with culture-based identification often differ from studies using 16S rRNA gene sequences to assign taxonomy, with the later often revealing a much greater diversity (15). Shade et al. (16) studied the same soil samples using culture and 16S rRNA gene sequencing techniques. These authors reported that cultured organisms were often low, or even absent, from 16S rRNA community profiles. This is similar to the present study, which revealed many more-abundant genera than was evident from previous culture-based studies, although most cultured genera were still detected. While one of the two most dominant genera of our study (*Aggregatibacter* spp.) was not reported by Machado et al. (40), their study was of uterine bacteria while the present study was of vaginal bacteria. However, given the location of the uterus relative to the vagina, it is likely that microbes enter the uterus via the vagina. In fact, during gestation this “ascending infection” in humans is hypothesized to be an important feature of pre-term birth (41).

The relative dominance of *Aggregatibacter* spp. and *Streptobacillus* spp. is interesting and draws parallels to the dominance of lactobacilli often seen in the human vagina (1). *Aggregatibacter* spp. have previously been observed at very low levels in samples of the human vagina (42), and described members include important human pathogens such as *A. aphrophilus* and *A. actinomycetemcomitans* that have been linked to periodontal disease, infective (HACEK) endocarditis, and brain abscess formation (42, 43). Their high relative abundance within the vaginal tracts of livestock may be facilitated by the ability of some members of this genus to adhere to collagen (44), which is a major component of the vaginal wall tissue in cows, and ewes, along with humans. In ewes, collagen accounts for up to 50% of ewe vaginal tissue (45, 46). While it is interesting to note that observed changes in human vaginal microbiota associated with menopause (47) take place at a time when the structure of collagen in the human vagina is also changing (48), the role of collagen in curating the vaginal microbiota is uncertain. The compositional structure and distribution of vaginal collagen in ewes is similar to that of humans and total collagen content is comparable (46). *Streptobacillus* spp. have also been observed in the human vagina (49). Much of what is known about this genus is based on *S. moniliformis*, the etiological agent of rat bite fever, which until recently was the only described species (50). Because of the paucity of information presently available on the Streptobacilli it is difficult to speculate on its role or niche within the ewe or cow vagina.

*Lactobacillus* spp. were prevalent, but at low relative abundances among cow and ewe vaginal microbiota. This is consistent with previously reported culture-driven results of the cow vagina (6–8). *Lactobacillus delbrueckii* was one *Lactobacillus* species detected in both cow and ewe vaginas (Table 2), which has previously been isolated from the cow vagina (8). The limited abundance of lactobacilli observed previously using culture-based studies had led Rodriguez et al. (8) to suggest that *Lactobacillus* spp. have a restricted role in the cow vagina.

The near-neutral pH observed herein is consistent with low *Lactobacillus* spp. abundance, as this genera is known to be able to produce large quantities of lactate as a metabolic by product (51), and is generally credited with creating low vaginal pH in women. Interestingly, the cow and ewe vaginal microbial communities share several notable genera, namely *Sneathia* spp., *Porphyromonas* spp., *Prevotella* spp., and a low abundance of *Lactobacillus* spp., with the CST IV described for humans by Ravel et al. (1). This CST corresponded with the highest vaginal pH in women, with a mean of 5.3. Manes et al. (14) reported pH means between 7.0 and 7.6 in ewes prior to synchronization with intravaginal sponges, although they reported a mean as low as 6.8 about 53 h after sponge removal at breeding. Beckwith-Cohen et al. (52) reported a range of 5.52–8.60 for cattle vaginal pH in the literature, and found that the mean vaginal pH of Israeli Holstein multiparous cows was 7.35. The mean cow and ewe pHs of 7.3 and 6.7, respectively, from the present study are comparable to these values.

In conclusion, 16S rRNA sequencing of cow and ewe vaginal ectocervicovaginal lavages revealed that cow and ewe vaginal microbiota are unique from previously described vaginal microbial ecosystems, though similar to one another. Cow microbiota exhibited greater diversity compared to the ewe microbiota, and both differed from humans and non-human primates. Bacteroidetes, Fusobacteria, and Proteobacteria were determined to be the dominant phyla. Archaea and lactobacilli, while prevalent, were not abundant. Culture methods previously employed likely misidentified the most abundant species, with organisms such as *Staphylococcus* spp. and *Streptococcus* spp. detected at very low abundance. The two most abundant members of the cow and ewe vaginal microbiota in the present study were *Aggregatibacter* spp. and *Streptobacillus* spp. It was confirmed that *Lactobacillus* spp., in contrast to the human vaginal microbiota, are not an abundant genera. The near-neutral pH observed in both cows and ewes is consistent with the low abundance of *Lactobacillus* spp. detected. However, it remains to be tested whether the taxa present in livestock vaginal systems are more prevalent or abundant during phases of the estrus cycle, affect reproductive performance, or contribute to perinatal colonization. Considering the great amount of diversity and different taxa identified using 16S rRNA sequencing, it would be valuable to explore these avenues with culture-independent analyses.

## Conflict of Interest Statement

The authors declare that the research was conducted in the absence of any commercial or financial relationships that could be construed as a potential conflict of interest.

## Supplementary Material

The Supplementary Material for this article can be found online at http://www.frontiersin.org/Journal/10.3389/fvets.2014.00019/abstract

## Footnotes

^1^http://www.mothur.org

^2^http://cran.r-project.org/web/packages/gplots/index.html
