# Supplementary material for: Characterization of the Vaginal Microbiota of Ewes and Cows Reveals a Unique Microbiota with Low Levels of Lactobacilli and Near-Neutral pH
Source: Front Vet Sci. 2014 Oct 15;1:19. doi: 10.3389/fvets.2014.00019 (PMC4672155; doi:10.3389/fvets.2014.00019)
Supplement: Supplementary file 1 [file Data_Sheet_1.PDF]

Supplementary Data

Supplementary Tables

Table S1 Shannons Diversity Measures And Comparison To Cow And Ewe Vaginal Microbiota

Host	Mean	Median	Std. Dev.	Shapiro-Wilk (p)[1]	p (Ewes)	p (Cows)
Ewe	2.87	2.97	1.16	0.130	N/A	0.046*
Cow	3.64	3.93	0.96	0.047†	0.046*	N/A
Human	0.64	0.37	0.59	0.011†	<0.001*	<0.001*
Chimpanzee	2.48	2.51	0.92	0.442	0.312	0.004*
Baboon	2.64	2.82	0.35	0.021†	0.790	0.013*
Howler	2.28	2.58	0.69	0.311	0.176	0.001*
Black Colobus	2.63	2.70	0.58	0.586	0.514	0.016*
Red Colobus	2.45	2.63	1.24	0.098	0.485	0.013*
Mangabey	2.61	2.51	0.53	0.875	0.454	0.013*
Vervet	1.37	1.26	0.56	0.152	<0.001*	<0.001*
Lemur	2.70	2.67	0.22	0.377	0.544	0.011*

1. **Data determined to be not normally distributed are indicated with '†'. [2.] Significant differences between ewe or cow diversity were assessed by two sample t-test for normally distributed data and by Wilcoxin-Mann-Whitney test for data that was not normally distributed. Significance was assessed at p <0.05 and is indicated with a '*'.**

Supplementary Figure 1

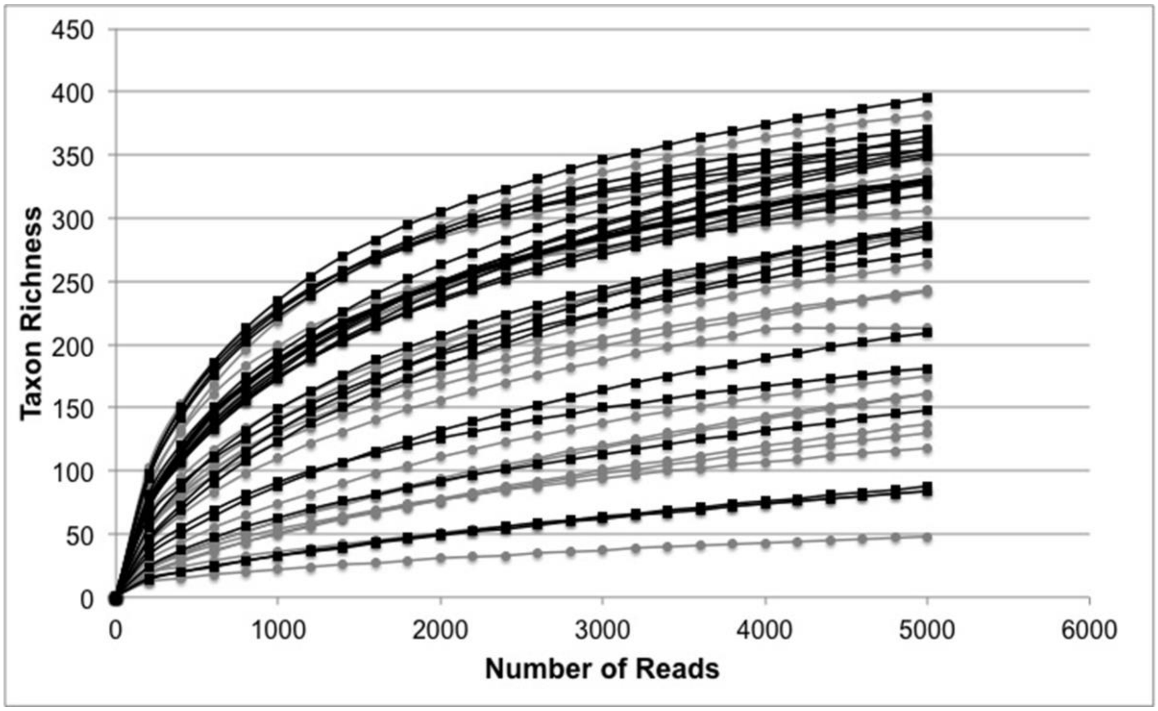

Fig S1. Rarefaction Curve of Detected Taxa in Ewe and Cow Vaginal Tracts
Curves from cow vaginal microbiota are shown in black, and ewes in grey.
